# Development and evaluation of a food environment survey in three urban environments of Kunming, China

**DOI:** 10.1186/1471-2458-14-235

**Published:** 2014-03-06

**Authors:** Jenna Hua, Edmund Seto, Yan Li, May C Wang

**Affiliations:** 1 School of Public Health, University of California, Berkeley, CA, USA; 2 School of Public Health, University of Washington, Seattle, WA, USA; 3 Kunming Medical University, Kunming, China; 4 Fielding School of Public Health, University of California, Los Angeles, CA, USA

**Keywords:** Food environment, Nutrition, Construct validity, Survey, Neighborhood, Adolescent, Kunming, China

## Abstract

**Background:**

Given the rapid pace of urbanization and Westernization and the increasing prevalence of obesity, there is a need for research to better understand the influence of the built environment on overweight and obesity in world’s developing regions. Culturally-specific food environment survey instruments are important tools for studying changing food availability and pricing. Here, we present findings from an effort to develop and evaluate food environment survey instruments for use in a rapidly developing city in southwest China.

**Methods:**

We developed two survey instruments (for stores and restaurants), each designed to be completed within 10 minutes. Two pairs of researchers surveyed a pre-selected 1-km stretch of street in each of three socio-demographically different neighborhoods to assess inter-rater reliability. Construct validity was assessed by comparing the food environments of the neighborhoods to cross-sectional height and weight data obtained on 575 adolescents in the corresponding regions of the city.

**Results:**

273 food establishments (163 restaurants and 110 stores) were surveyed. Sit-down, take-out, and fast food restaurants accounted for 40%, 21% and 19% of all restaurants surveyed. Tobacco and alcohol shops, convenience stores and supermarkets accounted for 25%, 12% and 11%, respectively, of all stores surveyed. We found a high percentage of agreement between teams (>75%) for all categorical variables with moderate kappa scores (0.4-0.6), and no statistically significant differences between teams for any of the continuous variables. More developed inner city neighborhoods had a higher number of fast food restaurants and convenience stores than surrounding neighborhoods. Adolescents who lived in the more developed inner neighborhoods also had a higher percentage of overweight, indicating well-founded construct validity. Depending on the cutoff used, 19% to 36% of male and 10% to 22% of female 16-year old adolescents were found to be overweight.

**Conclusions:**

The prevalence of overweight Chinese adolescents, and the food environments they are exposed to, deserve immediate attention. To our knowledge, these are the first food environment surveys developed specifically to assess changing food availability, accessibility, and pricing in China. These instruments may be useful in future systematic longitudinal assessments of the changing food environment and its health impact in China.

## Background

The rates of obesity among children and adults in the United States are alarming [1,2] and are responsible for staggering medical costs [3]. Elsewhere, the World Health Organization has declared obesity a global epidemic [4]. Considerable research has been conducted on the complex etiology of obesity, with environmental factors now thought to play an important role in influencing physical activity and diet [5-7]. Given the rapid pace of environmental change, urban development, and Westernization that is occurring in second and third world countries, there is great urgency to better understand the effect of the built environment on obesity and its behavioral risk factors in developing regions of the world. Indeed, in China, obesity rates among children have tripled in just two decades, from 1982 to 2002, reaching rates comparable to those in Western countries [8]. While some well-established larger cities in China already have high rates of obesity [8,9], medium sized cities that are still rapidly developing and currently have relatively lower rates of obesity are opportune sites for place-based research to understand the associations between the changing built environment and obesity. According to epidemiological studies conducted in one such medium sized Chinese city, Kunming, in 2008, it was estimated that 26% of the adults were overweight [10]. Moreover, in Kunming, the childhood obesity rate in 2008 was 36% higher than it was in 1996, and three times higher than it was found to be in 1986 [11].

Chinese adolescents who grew up in the 1990s during a period of rapid social and environmental change had increasing access to Western fast food and exposure to new technologies and media that market such food, including the Internet and smartphones. Nearly all were born under China’s one-child policy, and were the center of the attention of two generations, giving them the ability to concentrate on their education, but also enormous pressure to meet their parents’ and grandparents’ high expectations. At the same time, students at this age are highly susceptible to peer pressure [11-15], which influences their dietary patterns and other aspects of their behavior.

Numerous studies have reported associations between food-related aspects of the environment and obesity risk [16-20]. In the U. S. and other Western countries, this research has largely relied on the existence of business, land use, and tax record databases from governmental and commercial sources to characterize the food environment [17,21]. However, similar databases are not readily available for rapidly developing countries like China. Moreover, even if such databases were available, their validity would need to be assessed using field surveys.

Field survey instruments can be used to obtain detailed food environment data and to ground-truth food establishment databases. Such instruments allow for quantification of the number of food establishments in neighborhoods, as well as characterization of various aspects of the food offered by these establishments, including the availability and price of fresh and prepared foods that make up typical diets [22,23]. Field survey instruments need to be both reliable and valid. They need to be reliable in that ratings made by different surveyors need to be consistent. Also, among various validity measurements, construct validity is particularly important as it considers how the measures of the instrument relate to the overall theoretical hypotheses [24] – in our case, that the food environment is associated with weight status.

While reliable field survey instruments exist within the Western context [25-28], to our knowledge, no equivalent instruments currently exist to assess the food environment in China. Due to cultural differences in food availability and eating habits, survey instruments used in Western countries cannot be readily applied to China without modification. The development of a culturally appropriate and constructively valid survey instrument for characterizing the Chinese food environment is a critical step toward future studies on the evolution of food environments in a rapidly developing economy and its effects on the health of populations.

The objectives of this study are to: (1) develop a survey instrument for assessing the food environment in China; (2) assess its reliability in a rapidly developing Chinese city; (3) assess its construct validity for the hypothesis that the amount of fast food and packaged food in a Chinese community is positively associated with adolescent weight status; and (4) describe the density and types of food establishments in socioeconomically contrasting neighborhoods in such a city.

## Methods

### Survey instrument development

We developed two survey instruments: one to assess retail food stores and another to assess restaurants. To determine appropriate survey items, we reviewed the literature on food environment assessments via Google Scholar and PubMed, supplemented with a “snowballing” method to search for other relevant information. We found no existing tools appropriate for use in China, as the surveys that are used to assess food environment in United States were culturally inappropriate for the Chinese food environment, specifically the types of foods that are typically on restaurant menus and in food stores. However, the Nutrition Environment Measures Survey (NEMS) instrument used in the U.S. [25,28] provided ideas for conceptualizing the survey instruments, which we created with the assistance of our local Chinese collaborators at the Kunming Medical University. Both instruments (for store and restaurant assessment) were designed so that they could be completed within 10 minutes for a single food establishment by surveyors working independently, without disturbing the store or restaurant staff. The survey items and rationale for their inclusion are described in Tables 1 and 2. The two instruments were pretested for wording and content at restaurants, grocery stores and wet markets (open markets where stalls of fresh and prepared foods are sold by different vendors) in Kunming, and then finalized for pilot testing in three socio-demographically contrasting neighborhoods.

**Table 1:** Items captured by the survey instrument for assessing stores

**Item**	**Rationale/how measured**
Date	Essential food establishment data that allows for identification, geocoding, and time stamping.
Survey start time, survey end time	
Food establishment name	
Street address	
GPS reading	
Hours of operation	
**Type of store**	For classifying the stores (13 possible types). Categories are modified from those developed by the North American Industrial Classification System (NAICS) [29] used by the United States to classify food establishments. Specifically for Chinese context, even though both are selling cooked or prepared food, a deli is attached to a restaurant, and a take-out store is a stand-alone store. Check all that applied.
Wet market, supermarket, small market, convenience store, convenience store attached to a gas station, deli, take-out, bakery, street stand/cart, dessert/fruit juice, tobacco/alcohol shop and others	
**Store size**	Possibly a proxy for food variety. Estimated by assessing the length of a single floor tile and counting the number of perimeter tiles of each rectangular section of a store. (All stores had tiled floors)
Length and width	
**Types of items sold**	For assessing the availability of basic food items. Nine basic food items were assessed. Food item categories were determined by in-person store visit and consultation with local collaborators.
Soy products (raw), packaged foods, frozen meals, fresh cooked/prepared foods, basic grain products, processed/preserved dried meat and seafood, cooking oil, cleaned/easy-to-cook/combo meals, and cold desserts/ice cream	
**Indicator food items**	For assessing the availability of specific food items that are indicative of either a healthy or unhealthy diet. Fourteen indicator items were assessed. If available, shelf space and product location were further assessed (see below).
Salty snack, sweet snack, sweet drinks, alcohol, milk/yogurt, bottled water, powdered drinks, tea, instant noodles, pastry/baked goods, tofu products (packaged snack), fruits, vegetables and fresh meat/poultry	
For indicator food item that is present:	Shelf space assesses whether the indicator item occupies a significant amount of shelf space (significant or not). If certain products occupy more than half of the shelf space, it is counted as significant.
**Shelf space and product location**	Product location assesses whether the location of the item is in the front, middle, or back of the store.

**Table 2:** Items captured by the survey instrument for assessing restaurants

**Restaurant survey variables**	**Rationale/how measured**
Date	Essential food establishment data that allows for identification, geocoding, and time-stamping.
Survey start time, survey end time	
Food establishment name	
Street address	
GPS reading	
Hours of operation	
**Type of restaurant**	Categorization the restaurant. 14 possible types. Check all that applied. A distinction was made between establishments that sold prepared foods: if they had 5 or more seats, they were defined as restaurants, otherwise defined as a store.
Sit-down, take-out, western fast food, café, Chinese fast food, street stand/cart, food court, deli, bakery, bar, tea house, dessert, juice bar and other	
**Restaurant size**	Seating capacity in number of persons or number of tables.
Seating capacity	
**Type of food served**	Assess availability of certain types of foods that are common to this city. 11 types. Check all that applied.
Vegetarian, vegan, organic, dim-sum, seafood, noodles, regional cuisine, Muslim, buns/pancakes, deep-fried, and other	
**Drinks**	Assess availability of certain types of drinks that are common to this city. 10 types. Check all that applied.
None, soda, juice, alcohol, tea, coffee, bottled water, yogurt, flavored milk, and other	
Was a take-out menu available?	Yes/no
Was a flyer available?	Yes/no
**Advertisement**	Assesses whether the restaurant advertises their food. Check all that applied.
None, local TV station, phone directory, newspaper, and other	
Display of business license	Indication of potential food quality. Yes/no
Website	Indication of new forms of advertising. Yes/no
Nutrition information available	Yes/no
Signs encouraging overeating	e.g., all-you-can-eat, super-size, jumbo, extra-large descriptors on menu or signage. Yes/no
Promotions	e.g., low-carb, low-fat, low-cholesterol. Yes/no.
Portion size choices	Check if small, medium and large portions sizes are sold. Check all that applied.
**Price range**	Range of pricing for vegetable dishes, meat dishes, and other dishes.
Vegetable, meat, and others	

### Study area

Kunming is a rapidly developing city that is the capital of Yunnan province, located in the southwest region of China neighboring Tibet, Laos, Vietnam and Burma. Yunnan province has an ethnically diverse population consisting of Han and numerous ethnic minority groups. Because of its geographic location and recently recognized potential to serve as an international logistics center, Kunming has begun to link China with other Southeast Asian countries and is undergoing faster and more dramatic urbanization and environmental change than Beijing or Shanghai did during their peak development periods. Moreover, recent studies suggest that the prevalence of adult overweight and childhood obesity is becoming an increasing problem in Kunming [15,30]. Unless effective intervention strategies are applied, obesity-related chronic disease rates are expected to increase dramatically [10,31].

### Neighborhood selection

**Figure 1:**
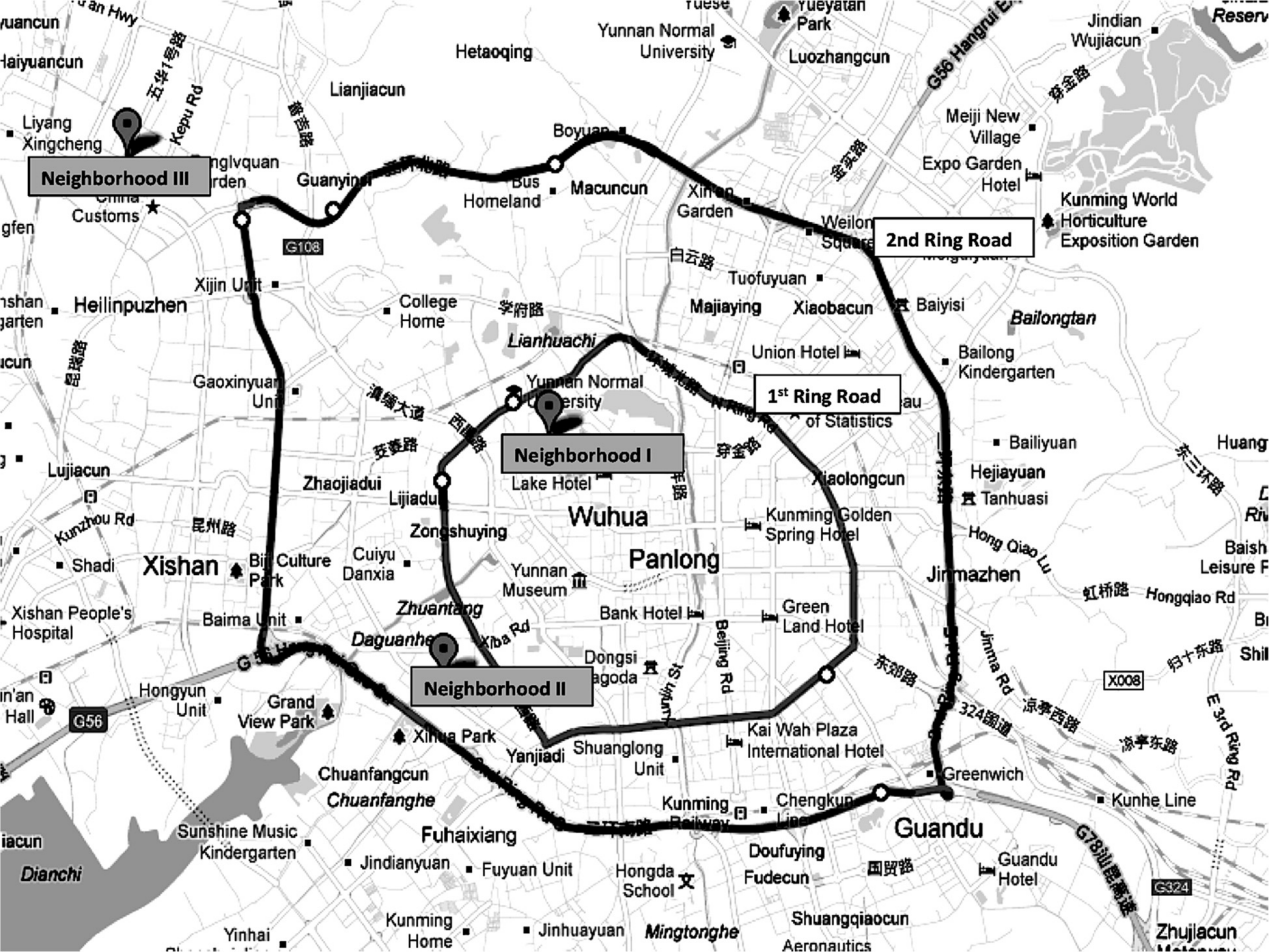
Neighborhood locations.

Three neighborhoods varying in distance from the center of Kunming were selected. Like many other Chinese cities, Kunming has concentric ring roads, which radiate from the city center and divide the neighborhoods of the city. Similar to the spatial urban development patterns in many Chinese cities, Kunming was developed according to proximity to the city center. Our study’s first neighborhood was situated within the first ring road, the second between the first ring road and the second ring road, and the third outside of the second ring road (Figure 1). These three neighborhoods vary in real estate prices, development histories, and land use characteristics (Table 3). In general, the more central areas of the city are older, with higher real estate and living costs, while the less costly outer areas consist of mixtures of urban slums, migrant worker areas, and newer developments that are rapidly replacing older communities. Because socio-demographic data at the neighborhood level was unavailable, we used the average neighborhood real estate prices as a proxy. In each neighborhood, the food environment assessments were carried out on a 1-km stretch of street with visibly high food establishment density. After areas in each neighborhood with high food establishment densities were identified by searching key terms (food establishments, restaurants, food stores, convenience stores, supermarkets and wet markets) at Google and Baidu Maps, 3 streets with highest food establishment densities were identified visually and ranked from first choice to third choice. Distances of 1-km were measured using built-in rulers in Google and Baidu Maps. Prior to conducting surveys, researchers visited the identified streets in each neighborhood to verify the existence and density of the food establishments, and made the final selection of a 1-km stretch of street in each neighborhood.

**Table 3:** **Neighborhood characteristics**[32]

**Neighborhoods**	**Location**	**Average real estate price (RMB/m**^**2**^**) in 2010**	**Development history**	**Primary land uses**
Neighborhood I (Wenlin)	Within the 1^st^ ring	13,000	Oldest	Residential, higher education site nearby
Neighborhood II (Ankang)	Between the 1^st^ and the 2^nd^ ring	9,000	Newer	Residential
Neighborhood III (Shangyuan)	Outside the 2^nd^ ring	7,500	Newest	Residential, higher educational institution nearby

### Reliability

To assess the inter-rater reliability of the instrument, two pairs of trained student researchers, attempted to survey all food stores and restaurants on each of the pre-selected 1-km stretches of street in the three neighborhoods. Surveys were conducted on weekdays between 10 am and 6:30 pm in July of 2011, with the two pairs of researchers surveying different neighborhoods on a given day to blind each pair to the other pair’s activities. If there was any discrepancy between the two researchers within a pair, both returned to the store or restaurant to make another observation and come to a mutual decision before completing the survey instrument. Having two researchers in a team permitted more efficient and accurate data collection in the field. The student researchers also measured the geographic location of each food establishment by using a GPS unit (Garmin 62S). This paper did not use the geographic location data collected; however, these data may be useful in the future to validate web-based secondary data sources on the food environment such as Google and Baidu Maps. The observations of the two pairs of researchers were compared using appropriate statistical methods as described below.

### Evaluation/validity

To evaluate and assess the construct validity of the instrument, measured heights and weights, as well as approximate home addresses of 575 adolescents from year 2011 were obtained from a local high school. These data were from the high school’s routine annual physical assessments of students. The selected high school was located between the first ring and second ring road, but has a wide catchment for students, who lived in various regions of the city. Home addresses were identified by neighborhood, which allowed us to categorize each adolescent’s home location in terms of the ring roads. Subjects with missing addresses were excluded from the analysis. Using both the data from the food environment surveys and this cohort’s data, we determined the extent to which the prevalence of western-style fast food restaurants and convenience stores (both tending to sell high fat and high caloric foods) is associated with higher prevalence of adolescent overweight and obesity. Adolescents’ weights, heights and approximate home addresses were recorded by the researchers without personal identifiers. Use of these de-identified data was approved by the Ethics Committees at Kunming Medical University and the University of California, Berkeley.

The weight statuses of the 575 adolescents were determined by calculating their body mass index (BMI) using their weight in kilograms divided by height in meters, squared. The BMIs were categorized into underweight, normal, overweight and obese. Because there was no single gold standard for BMI criteria for a Chinese population, we used five different BMI cutoffs to demonstrate different distributions of overweight and obese in the cohort. The five BMI cutoffs included those established by the Capital Institute of Pediatrics (CIP) and Working Group on Obesity in China (WGOC), International Obesity Task Force (IOTF), World Health Organization (WHO) and Center for Disease Control (CDC) [33-41]. Additionally, IOTF has two cutoffs, one specifically for Asians. Mean BMI and percentage of overweight adolescents were tabulated by different ring road locations for different genders and ages.

### Data analysis

Differences in counts of different types of food establishments and numbers of overweight or obese by neighborhood were assessed via Fisher’s exact test. For comparisons between dichotomous variables, chi-square test was used. The inter-rater reliability of categorical variables was assessed by calculating the percentage of agreement and Cohen’s kappa statistic:

$$\%\;\mathrm{agreement}=\left(A/N\right)\times 100$$

$$\kappa =\frac{\mathrm{Pr}\left(A\right)-\mathrm{Pr}\left(E\right)}{1-\mathrm{Pr}\left(E\right)}$$

where *A* is the number of times an item was categorized similarly by the two teams, and N is the number of stores/restaurants survey by both teams; and kappa is the probability of agreement Pr(*A*), adjusted by the probability of chance agreement Pr(*E*). Differences in the means of continuous variables estimated by the two teams were assessed with Student’s t-test. All tests used statistical significance level of p value less or equal to 0.05, and data analyses were conducted using STATA 11.2 (StataCorp, College Station, TX, 2012).

## Results

### Neighborhood comparison

A total of 273 food establishments including 163 restaurants and 110 retail stores were located on the 3 pre-selected 1-km stretches of street; data were obtained by both teams on 141 restaurants and 84 retail food stores, and these data were used to calculate inter-rater reliability. The unmatched 48 food establishments that were surveyed by only one of the teams were attributed to food establishments that were not open at the time of survey, as well as food establishments located at the intersection of the selected survey streets and adjacent streets. Sit-down restaurants accounted for almost 40% of restaurants, while take-out restaurants accounted for 21%. Fast food restaurants (both Western and Chinese) accounted for 19% of restaurants.

Among retail food stores, tobacco and alcohol shops accounted for nearly 25% of all stores, while take-out stores, convenience stores, and supermarkets accounted for 14%, 12% and 11% of all stores surveyed, respectively. In addition, there were just two wet markets (open markets that sell fresh produce and meats) in the three neighborhoods surveyed.

With only one street in each of the three neighborhoods sampled, our main objective was not to explore the relationship between neighborhood characteristics and food establishment counts. Nevertheless, we noted that the types and distributions of food establishments varied among the three neighborhoods (Tables 4 and 5). A restaurant or store could be classified into more than one category. For example, a sit-down restaurant selling western fast food would be classified as a sit-down restaurant and also a western fast food outlet. Neighborhood I (the oldest and most expensive neighborhood in the center of the city) had the highest restaurant count, while Neighborhood III (the newest and least expensive neighborhood on the city outskirts) had the second highest restaurant count. Interestingly, the neighborhood that had the highest restaurant count (Neighborhood I) also had the lowest store count. It was also observed that that in the city center, western-style fast food restaurants were prevalent (13 in Neighborhood I vs. 0 and 2 in Neighborhoods II and III respectively), while on the city outskirts, Chinese-style fast food restaurants were relatively more common than western-style fast food restaurants (9 in Neighborhood I vs. 15 and 7 in Neighborhoods II and III). Further, bars and cafes serving snacks/pastries were much more prevalent in Neighborhood I than in the other neighborhoods.

**Table 4:** Distribution of restaurants by neighborhood

			**Neighborhood**					
**Restaurant type**	**Total count**^**δ**^	**%**	**I**		**II**		**III**	
			**Count**	**%**	**Count**	**%**	**Count**	**%**
Sit-down	95	39.3	34	29.3	24	44.4	37	51.4
Take-out	52	21.5	20	17.2	12	22.2	20	27.8
Chinese fast food	31	12.8	9	7.8	15	27.8	7	9.7
Western fast food	15	6.2	13	11.2	0	0	2	2.8
Café (snacks/pastries)	14	5.8	12	10.3	0	0	2	2.8
Other	13	5.4	9	7.8	1	1.9	3	4.2
Bar	12	5	12	10.3	0	0	0	0
Tea House	6	2.5	4	3.5	1	1.9	1	1.4
Bakery	2	0.8	2	1.7	0	0	0	0
Food court	1	0.4	1	0.9	0	0	0	0
Deli	1	0.4	0	0	1	1.9	0	0
Total	242	100	116	100	54	100	72	100

^**δ**^Categories are not mutually exclusive and a restaurant/store may be counted in more than one category.

**Table 5:** Distribution of retail food stores by neighborhood

			**Neighborhood**					
**Store type**	**Total count**^**δ**^	**%**	**I**		**II**		**III**	
			**Count**	**%**	**Count**	**%**	**Count**	**%**
Tobacco & alcohol	20	23.8	5	26.3	13	31	2	8.7
Take-out	12	14.3	0	0	8	19.1	4	17.4
Convenience store	10	11.9	5	26.3	3	7.1	2	8.7
Supermarket	9	10.7	1	5.3	4	9.5	4	17.4
Other	8	9.5	2	10.5	6	14.3	0	0
Bakery	6	7.1	2	10.5	3	7.1	1	4.4
Dessert/fruit juice	5	6	0	0	0	0	5	21.7
Small market	4	4.8	2	10.5	1	2.4	1	4.4
Deli	4	4.8	0	0	2	4.8	2	8.7
Newspaper stand, street stand/cart	4	4.8	2	10.5	0	0	2	8.7
Wet market	2	2.4	0	0	2	4.8	0	0
Total	84	100	19	100	42	100	23	100

^**δ**^Categories are not mutually exclusive and a restaurant/store may be counted in more than one category.

### Characteristics of foods available

Selected characteristics of the most common foods offered by restaurants and stores are summarized in Tables 6 and 7. In restaurants, meat dishes tended to be more expensive than vegetable dishes, except at western fast food restaurants. Western fast food restaurants were more likely to offer deep-fried foods than Chinese sit-down, take-out, or even Chinese fast food restaurants. Interestingly, they were also more likely to offer dim-sum (bite-sized foods such as Chinese dumplings) than the other 3 common restaurant types. Chinese fast food consisted mainly of regional cuisine and noodle establishments. In terms of beverages, soda was available at most restaurants whereas bottled water was seldom available. Alcohol of various sorts was available at most restaurants except at sit-down restaurants. Most of the restaurants did not participate in any type of advertising although 3-13% had websites. Messages that encourage overeating such as “all-you-can-eat” promotions were visibly absent from the restaurants surveyed.

**Table 6:** Characteristics of four restaurant types

	**Restaurant type**			
	**Sit-down**	**Take-out**	**Western fast food**	**Chinese fast food**
Number of establishments	100	54	16	34
Seating capacity, mean (SD) persons	81.6 (92.6)	57.0 (77.6)	96.4 (95.8)	103 (128)
Type of food served				
Vegetarian, vegan, or organic	1%	2%	6%	0%
Dim-sum	31%	28%	69%	24%
Seafood	17%	9%	6%	26%
Noodles	29%	35%	19%	38%
Regional cuisine	24%	20%	0%	44%
Muslim	1%	2%	0%	3%
Buns/pancakes	10%	13%	0%	32%
Deep-fried	22%	20%	38%	24%
Drinks				
None	15%	26%	0%	21%
Soda	74%	61%	100%	62%
Juice	75%	67%	88%	62%
Alcohol	6%	35%	75%	62%
Tea	47%	44%	75%	35%
Coffee	25%	20%	81%	6%
Bottled water	9%	11%	0%	12%
Yogurt	12%	6%	31%	12%
Flavored milk	15%	13%	38%	9%
Take out menu available	8%	11%	31%	3%
Flyer available	4%	0%	6%	6%
Advertisement				
None	92%	94%	75%	97%
Local TV station	4%	4%	6%	3%
Phone directory	4%	2%	6%	3%
Newspaper	1%	0%	0%	3%
Display of business license	88%	89%	75%	97%
Website	4%	4%	13%	3%
Nutrition information available	0%	0%	0%	0%
Signs encouraging overeating	0%	0%	0%	0%
Promotions	0%	0%	0%	0%
Portion size choices	37%	41%	25%	35%
Prices (lowest priced)				
Vegetable, mean (SD) RMB	2.31 (10.2)	0.93 (2.32)	8.25 (24.4)	1.13 (2.31)
Meat, mean (SD) RMB	3.71 (8.35)	2.50 (6.38)	5.75 (13.3)	2.24 (3.92)

**Table 7:** Characteristics of four food store types

	**Food store type**			
	**Wet market**	**Supermarket**	**Small market**	**Convenience store**
Number of establishments	2	9	4	10
Store size, m^2^	8100 (2121)	233 (360)	59.4 (42.6)	37.4 (48.0)
Types of items sold				
Soy products	100%	0%	0%	10%
Packaged foods	100%	67%	0%	30%
Frozen meals	0%	11%	0%	10%
Fresh cooked/prepared foods	100%	0%	0%	0%
Basic grain products	100%	56%	0%	20%
Processed/preserved dried meat and seafood	100%	33%	0%	20%
Cooking oil	100%	56%	0%	20%
Cleaned/easy-to-cook/combo meals	0%	0%	0%	0%
Cold dessert/ice cream	100%	78%	0%	30%
Indicator food items				
Salty snack	0%	100%	0%	60%
Sweet snack	0%	100%	0%	60%
Sweet drinks	100%	100%	0%	67%
Alcohol	0%	100%	0%	80%
Milk/yogurt	100%	89%	0%	50%
Bottled water	100%	89%	0%	70%
Powdered drinks	0%	67%	0%	70%
Tea	100%	44%	0%	10%
Instant noodles	0%	78%	0%	60%
Pastry/baked goods	100%	33%	0%	50%
Tofu products	100%	33%	0%	0%
Fruits	100%	0%	75%	0%
Vegetables	100%	0%	0%	0%
Fresh meat/poultry	100%	0%	0%	0%

The survey revealed the wide variety of food stores available in Kunming (Table 7). Wet markets were few in number but, when present, they typically carried fresh fruits and vegetables and meat. In contrast, neighborhood supermarkets and convenience stores mostly sold packaged and processed foods and beverages; few carried fresh fruits, vegetables and meats.

### Reliability

Inter-rater reliability between the two survey teams for restaurant and store-related categorical variables is reported in Tables 8 and 9, respectively. In general, the percentage of agreement was high (>75%) for all categorical variables, and the average kappa statistics ranged from 0.162 to 0.648. Most kappa scores were between 0.4 to 0.6, indicating moderate agreement. The poorer kappa statistics tended to be associated with survey items that were not immediately observable (i.e., required questioning a person from the restaurant or store), such as whether the restaurant advertises or has a website. Thus, these person-reported surveys responses may reflect the varying degrees of knowledge of the restaurant staff versus managers or owners. We found no statistically significant differences between teams for any of the continuous variables (Table 10).

**Table 8:** Inter-rater reliability for categorical items: restaurants

**Restaurant**	**Team 1**	**Team 2**	**% agreement**	**Kappa**	**SE**
**Type of restaurant**					
Sit-down	101	100	83.9	0.6	0.084
Take-out	82	54	73.4	0.5	0.077
Western fast food	6	16	93.0	0.5	0.073
Chinese fast food	5	34	79.7	0.2	0.051
Food court	1	1	100.0	1.0	0.084
Deli	4	1	97.9	0.4	0.067
Bakery	2	2	100.0	1.0	0.084
Bar	37	12	82.5	0.4	0.068
Tea house	2	6	95.8	0.2	0.072
Dessert	7	14	88.1	0.1	0.078
Other	11	14	88.1	0.3	0.083
**Average**	**23**	**23**	**89.3**	**0.5**	**0.075**
**Type of food served**					
Vegetarian	0	1	99.3	0.0	N/A
Dim-Sum	25	34	86.7	0.6	0.082
Seafood	22	17	88.1	0.5	0.083
Noodles	31	29	83.2	0.5	0.084
Regional cuisine	12	25	86.7	0.4	0.077
Muslim	3	2	97.9	0.4	0.082
Buns/pancakes	18	12	93.0	0.6	0.082
Deep-fried foods	35	25	81.8	0.5	0.082
Other	18	30	67.8	0.1	0.080
**Average**	**18**	**19**	**87.2**	**0.4**	**0.081**
**Drinks**					
N/A	43	47	90.2	0.8	0.083
Soda	80	82	87.4	0.7	0.084
Juice	86	81	85.3	0.7	0.083
Alcohol	62	67	88.1	0.8	0.083
Tea	52	54	84.6	0.7	0.084
Coffee	31	29	88.8	0.7	0.084
Bottled water	27	11	83.2	0.3	0.074
Yogurt	21	12	86.7	0.4	0.080
Flavored milk	31	17	80.4	0.3	0.079
Other	8	3	93.7	0.2	0.074
**Average**	**44**	**40**	**86.9**	**0.5**	**0.081**
Takeout menu	76	11	53.1	0.1	0.042
Flyers	41	6	75.3	0.2	0.050
**Advertised**					
N/A	115	134	82.5	0.3	0.069
Local phone directory	21	4	86.7	0.2	0.049
Newspaper	2	1	97.9	0.0	0.079
Local TV station	8	4	94.4	0.3	0.079
Other	6	4	93.0	0.0	0.082
**Average**	**30**	**29**	**90.9**	**0.2**	**0.072**
Business license	109	104	85.3	0.6	0.083
Website available	6	7	93.2	0.1	0.084
Nutrition information	1	0	99.3	0.0	N/A
Overeating	2	0	98.6	0.0	0.000
Special options	9	0	93.7	0.0	0.000
**Portion choices**					
N/A	98	104	83.2	0.6	0.083
Small	44	39	83.9	0.6	0.083
Medium	25	12	86.7	0.4	0.077
Large	43	39	81.8	0.6	0.083
**Average**	**53**	**49**	**83.9**	**0.5**	**0.082**

**Table 9:** Inter-rater reliability for categorical items: food stores

**Food store**	**Team 1**	**Team 2**	**% agreement**	**Kappa**	**SE**
**Type of store**					
Wet market	2	2	100.0	1.0	0.111
Supermarket	9	9	95.1	0.8	0.111
Small market	3	4	96.3	0.6	0.110
Convenience store	2	10	87.7	0.1	0.080
Deli	4	4	100.0	1.0	0.111
Take-out	2	12	87.7	0.3	0.074
Bakery	6	6	100.0	1.0	0.111
Newspaper stand, street stand/cart	5	4	96.3	0.7	0.110
Dessert/fruit juice	5	5	100.0	1.0	0.111
Tobacco and alcohol shop	13	20	86.4	0.6	0.107
Other	28	8	70.4	0.2	0.084
**Average**	7	8	**92.7**	**0.6**	**0.102**
**Item sold**					
Tofu products	0	1	98.8	0.0	0.000
Packaged foods	10	9	96.3	0.8	0.106
Frozen dumplings/meals	1	2	98.8	0.7	0.105
Fresh cooked prepared foods	0	2	97.5	0.0	0.000
Basic grain products	6	7	96.3	0.7	0.111
Processed, preserved dried meat and seafood	10	6	95.1	0.7	0.107
Cooking oil	9	7	97.5	0.9	0.110
Ice cream, cold dessert	11	18	86.4	0.5	0.106
**Average**	**6**	**7**	**95.8**	**0.5**	**0.081**

**Table 10:** Reliability statistics for restaurant and store continuous variables

	**Team 1 mean (SD)**	**Team 2 mean (SD)**	**P**
**Restaurants**			
Seating capacity, persons	62.4 (84.0)	62.6 (90.6)	0.99
Vegetable (lowest priced), RMB	1.41 (3.14)	1.72 (8.61)	0.68
Meat (lowest priced), RMB	3.76 (10.6)	2.87 (7.79)	0.42
**Stores**			
Store size, m^2^	44.6 (111)	44.9 (136)	0.98

### Adolescent BMI distributions

**Figure 2:**
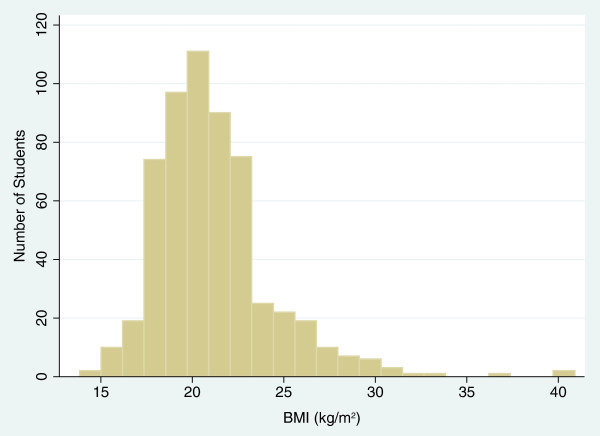
BMI distributions of 575 students.

**Figure 3:**
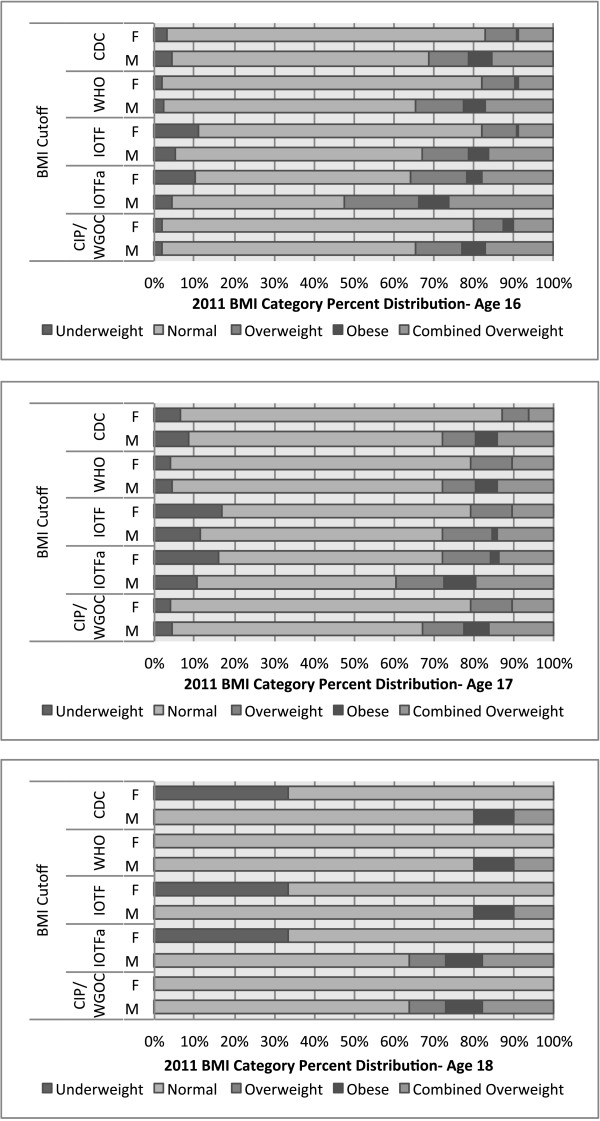
Percentage distributions of BMI categories among 5 different BMI cutoffs adjusting for gender and age for students aged 16 to 18.

The distribution of 575 adolescents’ BMIs is illustrated in Figure 2 with mean BMI of 21.1 kg/m^2^ and standard deviation of 3.2 kg/m^2^. The percentage distribution of BMI categories for five different cutoffs is reported in Figure 3 and Additional file 1. Mean BMIs and percentages of overweight adolescents at categorized locations, adjusted for gender and age, are reported in Tables 11 and 12. More than 85% of the students were between ages 16 and 17. At age 16, IOTF Asian cutoffs generated the highest percentage (35.6% for male and 21.8% for female) of overweight (combined overweight and obese) adolescents; WGOC cutoffs generated 21.0% for males and 11.1% for females; IOTF regular cutoffs generated 19.8% for males and 9.9% for females; WHO cutoffs generated 20.9% for males and 9.9% for females, and CDC cutoffs generated the lowest percentage (18.6% for males and 9.5% for females). There was higher prevalence of overweight and obesity in males than females. In terms of underweight, CIP’s below-5^th^-percentile cutoff and WHO cutoff generated 2.3% and 2.0% of underweight male and female adolescents, IOTF cutoff generated 6.2% for males and 12.3% for females, and CDC cutoff generated 5.1% for males and 3.6% for females.

**Table 11:** Numbers and percentage of cohort and their average BMIs in different regions of the city adjusting for gender and age

**Age, yr**	**Home location**											
	**Within 1st ring**				**Between the 1st and 2nd ring**				**Outside the 2nd ring**			
	**Male**		**Female**		**Male**		**Female**		**Male**		**Female**	
	**N (%)**	**Avg BMI (SD)**	**N (%)**	**Avg BMI (SD)**	**N (%)**	**Avg BMI (SD)**	**N (%)**	**Avg BMI (SD)**	**N (%)**	**Avg BMI (SD)**	**N (%)**	**Avg BMI (SD)**
13	0	0	0	0	0	0	0	0	0	0	1 (0.5)	19.3 (N/A)
14	0	0	0	0	0	0	2 (2.6)	24.7 (3.08)	0	0	1 (0.5)	22.9 (N/A)
15	0	0	3 (8.3)	20.1 (1.51)	1 (1.5)	19.7 (N/A)	5 (6.5)	21.3 (3.50)	4 (3.0)	19.5 (1.46)	11 (5.6)	20.7 (1.82)
**16**	28 (71.8)	**21.7 (3.58)**	30 (83.3)	***21.6 (3.11)***	45 (68.2)	**21.4 (3.97)**	58 (75.3)	***20.5 (2.78)***	93 (70.5)	**21.3 (3.83)**	152 (77.9)	***20.8 (2.55)***
**17**	11 (28.2)	**21.9 (2.46)**	3 (8.3)	***19.4 (1.24)***	18 (27.3)	**21.7 (4.45)**	11 (14.3)	***21.5 (2.71)***	28 (21.2)	**21.3 (3.33)**	28 (14.4)	***20.7 (2.70)***
18 and above	0	0	0	0	2 (3.0)	22.8 (2.84)	1 (1.3)	17.4 (N/A)	7 (5.3)	23.8 (7.43)	2 (1.0)	19.3 (0.03)
**Total**	39 (100)	**21.8 (3.27)**	36 (100)	***21.3 (2.95)***	66 (100)	**21.5 (4.01)**	77 (100)	***20.8 (2.86)***	132 (100)	**21.4 (3.95)**	195 (100)	***20.8 (2.51)***

Percentage of students aged 16-17 and their average BMIs in different regions of the city were **BOLD** as more than 85% of the students were between ages 16 and 17.

**Table 12:** Numbers and percentage of cohort that is overweight or obese in different regions of the city for various BMI cutoffs

**Age 14**												
**Home location**	**Within the 1st ring**				**Between the 1st and 2nd ring**				**Outside the 2nd ring**			
	**N = 0**		**N = 0**		**N = 0**		**N = 2**		**N = 0**		**N = 1**	
BMI cutoffs	M	%	F	%	M	%	F	%	M	%	F	%
CIP/WGOC	0	0	0	0	0	0	1	50.0	0	0	0	0.0
IOTFa	0	0	0	0	0	0	2	100.0	0	0	1	100.0
IOTF	0	0	0	0	0	0	1	50.0	0	0	0	0.0
WHO	0	0	0	0	0	0	1	50.0	0	0	1	100.0
CDC	0	0	0	0	0	0	1	50.0	0	0	0	0.0
**Age 15**												
**Home location**	**Within the 1st ring**				**Between the 1st and 2nd ring**				**Outside the 2nd ring**			
	**N = 0**		**N = 3**		**N = 1**		**N = 5**		**N = 3**		**N = 11**	
BMI cutoffs	M	%	F	%	M	%	F	%	M	%	F	%
CIP/WGOC	0	0	0	0	0	0	2	40.0	0	0	1	9.1
IOTFa	0	0	0	0	0	0	2	40.0	0	0	3	27.3
IOTF	0	0	0	0	0	0	1	20.0	0	0	1	9.1
WHO	0	0	0	0	0	0	2	40.0	0	0	1	9.1
CDC	0	0	0	0	0	0	1	20.0	0	0	1	9.1
**Age 16**												
**Home location**	**Within the 1st ring**				**Between the 1st and 2nd ring**				**Outside the 2nd ring**			
	**N = 28**		**N = 30**		**N = 45**		**N = 58**		**N = 93**		**N = 152**	
BMI cutoffs	M	%	F	%	M	%	F	%	M	%	F	%
CIP/WGOC	6	**21.4**	7	***23.3***	7	**15.6**	6	***10.3***	22	**23.7**	14	***9.2***
IOTFa	11	**39.3**	9	***30.0***	15	**33.3**	15	***25.9***	31	**33.3**	28	***18.4***
IOTF	5	**17.9**	7	***23.3***	6	**13.3**	5	***8.6***	22	**23.7**	12	***7.9***
WHO	6	**21.4**	7	***23.3***	7	**15.6**	5	***8.6***	22	**23.7**	12	***7.9***
CDC	5	**17.9**	7	***23.3***	6	**13.3**	5	***8.6***	20	**21.5**	12	***7.9***
**Age 17**												
**Home location**	**Within the 1st ring**				**Between the 1st and 2nd ring**				**Outside the 2nd ring**			
	**N = 11**		**N = 3**		**N = 18**		**N = 11**		**N = 28**		**N = 28**	
BMI cutoffs	M	%	F	%	M	%	F	%	M	%	F	%
CIP/WGOC	2	**18.2**	0	0	5	**27.8**	2	***18.2***	4	**14.3**	3	***10.7***
IOTFa	3	**27.3**	0	0	6	**33.3**	3	***27.3***	6	**21.4**	4	***14.3***
IOTF	2	**18.2**	0	0	5	**27.8**	2	***18.2***	3	**10.7**	3	***10.7***
WHO	2	**18.2**	0	0	5	**27.8**	2	***18.2***	3	**10.7**	3	***10.7***
CDC	2	**18.2**	0	0	5	**27.8**	2	***18.2***	3	**10.7**	1	***3.6***
**Age 18 and above**												
**Home location**	**Within the 1st ring**				**Between the 1st and 2nd ring**				**Outside the 2nd ring**			
	**N = 0**		**N = 0**		**N = 2**		**N = 1**		**N = 7**		**N = 2**	
BMI cutoffs	M	%	F	%	M	%	F	%	M	%	F	%
CIP/WGOC	0	0	0	0	1	50.0	0	0	1	14.3	0	0
IOTFa	0	0	0	0	1	50.0	0	0	1	14.3	0	0
IOTF	0	0	0	0	0	0	0	0	1	14.3	0	0
WHO	0	0	0	0	0	0	0	0	1	14.3	0	0
CDC	0	0	0	0	0	0	0	0	1	14.3	0	0

Percentage of students aged 16-17 and their average BMIs in different regions of the city were **BOLD** as more than 85% of the students were between ages 16 and 17.

### Evaluation/validity

Construct validity of the survey instruments is reported in Tables 11 and 12. There were 30 students with missing home addresses; therefore, a total of 545 students were included in the validity tabulation. In general, regardless of the cutoffs used, the percentages of overweight adolescents were highest for those who lived within the 1^st^ ring. And, those who lived between the 1^st^ and 2^nd^ ring had higher percentages of overweight than those who lived outside the 2^nd^ ring. Although the differences were small and only marginally statistically significant (Student’ t-test p = 0.077 to 0.131 between within 1^st^ and outside of 2^nd^ ring groups depending on which cutoff is used), there was consistency with differences in mean BMI between categories for students aged 16 to 17. The mean BMIs of those who lived at three locations were 21.7, 21.4 and 21.3 kg/m^2^ (SD 3.58, 3.97, and 3.83) for males, and 21.6, 20.5 and 20.8 kg/m^2^ (SD 3.11, 2.78, and 3.55) for females, respectively. This was also consistent with the distribution of western-style fast food restaurants and convenience stores in three neighborhoods, with the neighborhood within the 1^st^ ring having the highest count of western-style fast food restaurants and convenience stores illustrated in Tables 4 and 5. This provides indication of validity that the survey is adequately sampling food environments relevant to adolescent obesity.

## Discussion

The development and validation of culturally-specific food environment survey instruments is an important step towards the conduct of studies of changing food availability, access, and pricing in second and third world countries, where obesity rates are rising rapidly. Here, we present the results of an effort, motivated by the use of the NEMS instrument within the American context to study food environments, to develop and validate an instrument for surveying restaurants and food stores in a rapidly developing city in southwest China. Overall, both the restaurant and store food environment instruments were found to have excellent percentage of agreement and moderate kappa scores, as well as well-founded construct validity.

There are limitations to our survey. In particular, restriction to a 1-km stretch of street in only 3 neighborhoods is clearly not representative of all of China or even of all the neighborhoods of Kunming, and by limiting our surveys to 10 minutes, there were many aspects of the food environment that were not recorded. For example, we were unable to properly assess the prices of all food items. We were also unable to properly measure shelf space and instead, developed a rapid assessment technique that involved the counting of floor tiles that were measured at each store. However, our experience during rounds of pilot testing suggested that surveyor fatigue and store/restaurant’s unwillingness to cooperate significantly impacted the survey qualities when the surveys were longer than 10 minutes. Despite these limitations, our study of three socio-demographically contrasting neighborhoods provides a fairly rich glimpse of the types of food environment changes that may be occurring as a result of ongoing globalization and the introduction of western-style fast food into other countries’ food environments. Unhealthy qualities attributed to fast food were found in our study. In particular, high percentages of deep-fried foods, soda, and snack-like meals were found in restaurants and food stores, and prices of vegetables tended to be higher than those of meat products. Clearly, there is a need for more systematic longitudinal assessments of changing food environments in this cultural context, which could advance understanding of the influences of rapidly changing environments on non-communicable disease risk. Since the development of this instrument in 2011, we have conducted surveys in 2012 and 2013. While it is not the goal of this paper to describe longitudinal changes that may be occurring, as they are multi-faceted and complex, based on our preliminary analyses, the food environment instrument does seem to be sensitive in detecting certain key changes within neighborhoods such as changes in the numbers of sit-down and take-out restaurants, dessert shops, as well as convenience stores.

**Figure 4:**
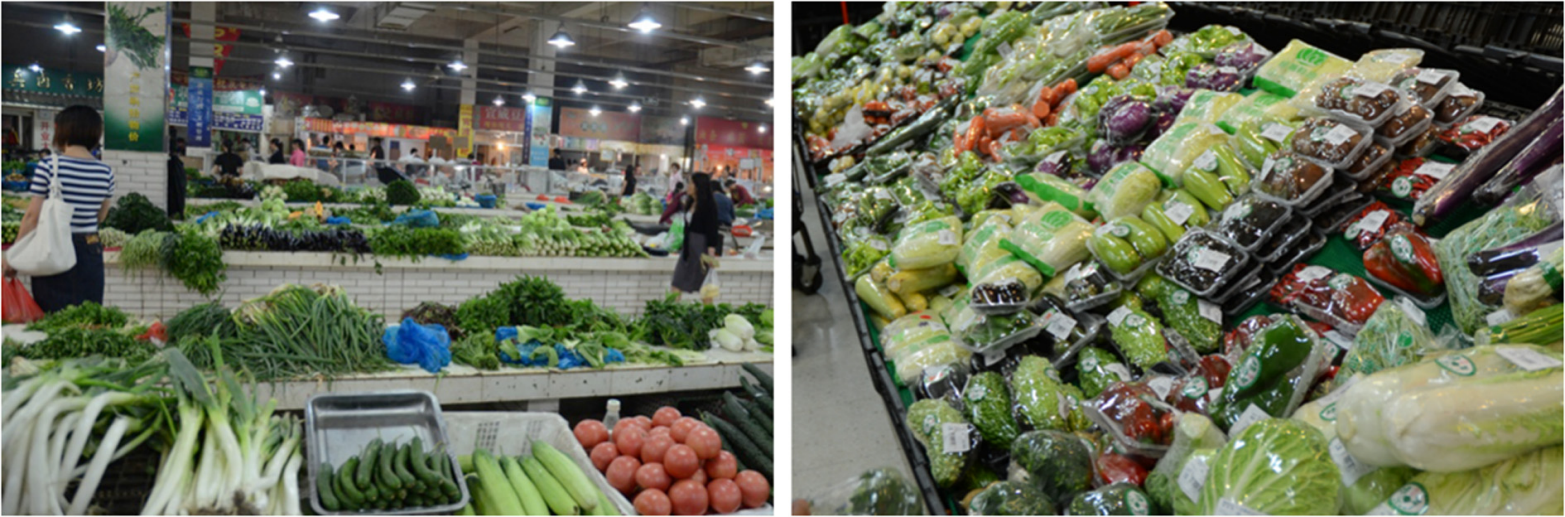
Vegetables sold in the wet market (left) compared to those sold in the mega-supermarket (right).

In contrast to the changes in the prevalence of certain foods that may be occurring with the proliferation of western fast food, some aspects of the food environment remain characteristically Chinese. For example, we found that traditional wet markets remain important places for people in the city to buy fresh fruits, vegetables and meat. In fact, none of the local supermarkets and convenience stores in our three neighborhoods sold these fresh foods. A point of concern is that supermarkets and convenience stores are ubiquitous, with one on nearly every street block, which makes packaged and processed foods and beverages more readily accessible than fresh food from the wet markets. We also observed that wet markets had limited operating hours. The wet market stalls are independently rented and operated, and hence may only be open during early morning and late afternoons, or only on certain days. This may affect food accessibility for some populations, such as workers and students. Further, the era of the wet market may be coming to an end. During our field study in Kunming we visited a few internationally owned ‘mega supermarkets.’ In the summer of 2011, there were three Wal-Mart and six Carrefour mega stores in the city. These mega stores all allocated a large proportion of their shelves to food products. Although we were not allowed by management to conduct our survey in their stores, we observed food delis that served many different types of cooked foods. Additionally, we observed aisle after aisle of packaged and fresh foods. Fresh fruits and vegetables were sold individually as well as in convenience packages – prewashed (Figure 4) and bundled for quick meal preparation. The prices were comparable to the wet markets, and the stores were packed with customers when we visited.

Our study is one of the few that present data on adolescent BMIs for middle-sized cities in western China. Adolescents who lived in the inner neighborhoods of Kunming tended to have higher percentage of overweight indicating the surveys were valid in assessing food environment – obesity relationships. Specifically, adolescents who live within the 1^st^ ring tended to have higher western-style fast food and packaged food exposures or unhealthy food environment exposures than those who live between the 1^st^ and 2^nd^ ring and those who live outside the 2^nd^ ring. While obesity is complex and affected by many factors such as built environment characteristics and socio-demographic status, even when unadjusted for confounding factors, differences in the food environment tended to be associated with observed differences in the rates of adolescent overweight and obese between neighborhoods. Additional research is needed to determine whether this association may be due to potential confounding factors (e.g., access to physical activity environments, environmental stress, etc.), rather than the food environment. As we consider future multivariate analyses of the associations between neighborhood-level differences and BMI, there may be value in considering BMI as a continuous variable in regression analyses to avoid loss of information through the tricotomization of BMI into underweight, normal, and overweight categories. Moreover, there would be considerable value in collecting actual data on dietary patterns as a mediator between the food environment and obesity. Ongoing research by our group aims to further explore the linkages between the food environment, dietary behavior, and weight status for adolescents. Nevertheless, our current findings are consistent with several studies that demonstrated that proximity to fast foods is positively associated with diet and higher BMI in Western contexts [23,42-50].

Different BMI cutoffs generated slight to moderate differences in the distributions of overweight and obese categories. IOTF Asian cutoff generated the highest percentages of overweight adolescents (35.6% for males and 21.8% for females) whereas CDC cutoffs only generated 18.6% for males and 9.5% for females, less than half of the IOTF Asian cutoff. In terms of underweight adolescents, CIP and WHO cutoffs generated around 2.0% of underweight males and females, whereas IOTF cutoff generated 6.2% for males and 12.3% for females, about three times and six times higher than the WHO percentage, respectively. Therefore, it is important for researchers to consider different cutoffs, and their use on particular populations. Choosing appropriate cutoffs will be extremely important in developing intervention strategies and making policy recommendations. Regardless of the BMI cutoffs used, however, we found the prevalence of overweight adolescents worrying, especially for male adolescents. Should these findings be reinforced by other larger studies in Chinese cities, action in the form of new obesity prevention strategies may be warranted to combat this problem.

## Conclusions

The rates of overweight and obesity among Chinese adolescents deserves immediate attention, and requires the development of reliable, valid, and culturally-appropriate instruments to track risk factors for obesity. To our knowledge, this is the first food environment survey instrument developed to specifically assess changing food availability, accessibility and pricing in China. Moreover, this is one of the few studies that provide insights into rates of adolescent overweight/obesity in a middle-sized city in western China. This instrument can be used for conducting systematic longitudinal assessments of the changing food environment in rapidly developing Chinese cities where there is an urgent need to monitor changing disease risk.

## Abbreviations

CIP: Capital Institute of Pediatrics; WGOC: Working Group on Obesity in China; IOTFa: International obesity task force Asian cutoff; IOTF: International Obesity Task Force; WHO: World Health Organization; CDC: Center for disease control.

## Competing interests

The authors declare that they have no competing interests.

## Authors’ contributions

All authors were involved in various stages of study design, survey development and validation. JH carried out the fieldwork and wrote the paper. JH and ES conducted the statistical analysis. ES, YL and MW oversaw the study progress and ensured the successful survey field deployment. All authors commented on drafts and approved the final text.

## Pre-publication history

The pre-publication history for this paper can be accessed here:

http://www.biomedcentral.com/1471-2458/14/235/prepub

## Supplementary Material

Additional file 1 **Percentage distribution of BMI categories among 5 different BMI cutoffs adjusting for gender and age.** Word document: this table was too large to be included in the main text. Click here for file
