# Supplementary material for: Development and evaluation of a food environment survey in three urban environments of Kunming, China
Source: BMC Public Health. 2014 Mar 6;14:235. doi: 10.1186/1471-2458-14-235 (PMC4016521; doi:10.1186/1471-2458-14-235)
Supplement: Additional file 1 — Percentage distribution of BMI categories among 5 different BMI cutoffs adjusting for gender and age. Word document: this table was too large to be included in the main text. [file 1471-2458-14-235-S1.docx]

| BMI Category | BMI Cutoff | | | | | | | | | | | | | | | | | | | |
| --- | --- | --- | --- | --- | --- | --- | --- | --- | --- | --- | --- | --- | --- | --- | --- | --- | --- | --- | --- | --- |
|  | CIP/WGOC | | | | IOTFa | | | | IOTF | | | | WHO | | | | CDC | | | |
|  | M | % | F | % | M | % | F | % | M | % | F | % | M | % | F | % | M | % | F | % |
| Age 13 | | | | | | | | | | | | | | | | | | | |  |
| Underweight | 0 | N/A | 0 | 0.0 | 0 | N/A | 0 | 0.0 | 0 | N/A | 0 | 0.0 | 0 | N/A | 0 | 0.0 | 0 | N/A | 0 | 0.0 |
| Normal | 0 | N/A | 1 | 100.0 | 0 | N/A | 1 | 100.0 | 0 | N/A | 1 | 100.0 | 0 | N/A | 1 | 100.0 | 0 | N/A | 1 | 100.0 |
| Overweight | 0 | N/A | 0 | 0.0 | 0 | N/A | 0 | 0.0 | 0 | N/A | 0 | 0.0 | 0 | N/A | 0 | 0.0 | 0 | N/A | 0 | 0.0 |
| Obese | 0 | N/A | 0 | 0.0 | 0 | N/A | 0 | 0.0 | 0 | N/A | 0 | 0.0 | 0 | N/A | 0 | 0.0 | 0 | N/A | 0 | 0.0 |
| Combined Overweight | 0 | N/A | 0 | 0.0 | 0 | N/A | 0 | 0.0 | 0 | N/A | 0 | 0.0 | 0 | N/A | 0 | 0.0 | 0 | N/A | 0 | 0.0 |
| Total | 0 | N/A | 1 | 100.0 | 0 | N/A | 1 | 100.0 | 0 | N/A | 1 | 100.0 | 0 | N/A | 1 | 100.0 | 0 | N/A | 1 | 100.0 |
| Age 14 | | | | | | | | | | | | | | | | | | | |  |
| Underweight | 0 | N/A | 0 | 0.0 | 0 | N/A | 0 | 0.0 | 0 | N/A | 0 | 0.0 | 0 | N/A | 0 | 0.0 | 0 | N/A | 0 | 0.0 |
| Normal | 0 | N/A | 2 | 66.7 | 0 | N/A | 0 | 0.0 | 0 | N/A | 2 | 66.7 | 0 | N/A | 1 | 33.3 | 0 | N/A | 2 | 66.7 |
| Overweight | 0 | N/A | 0 | 0.0 | 0 | N/A | 2 | 66.7 | 0 | N/A | 1 | 33.3 | 0 | N/A | 2 | 66.7 | 0 | N/A | 1 | 33.3 |
| Obese | 0 | N/A | 1 | 33.3 | 0 | N/A | 1 | 33.3 | 0 | N/A | 0 | 0.0 | 0 | N/A | 0 | 0.0 | 0 | N/A | 0 | 0.0 |
| Combined Overweight | 0 | N/A | 1 | 33.3 | 0 | N/A | 3 | 100.0 | 0 | N/A | 1 | 33.3 | 0 | N/A | 2 | 66.7 | 0 | N/A | 1 | 33.3 |
| Total | 0 | N/A | 3 | 100.0 | 0 | N/A | 3 | 100.0 | 0 | N/A | 3 | 100.0 | 0 | N/A | 3 | 100.0 | 0 | N/A | 3 | 100.0 |
| Age 15 | | | | | | | | | | | | | | | | | | | |  |
| Underweight | 0 | 0.0 | 0 | 0.0 | 0 | 0.0 | 0 | 0.0 | 0 | 0.0 | 0 | 0.0 | 0 | 0.0 | 0 | 0.0 | 0 | 0.0 | 0 | 0.0 |
| Normal | 5 | 100.0 | 18 | 85.7 | 5 | 100.0 | 16 | 76.2 | 5 | 100.0 | 19 | 90.5 | 5 | 100.0 | 18 | 85.7 | 5 | 100.0 | 19 | 90.5 |
| Overweight | 0 | 0.0 | 3 | 14.3 | 0 | 0.0 | 4 | 19.0 | 0 | 0.0 | 2 | 9.5 | 0 | 0.0 | 3 | 14.3 | 0 | 0.0 | 2 | 9.5 |
| Obese | 0 | 0.0 | 0 | 0.0 | 0 | 0.0 | 1 | 4.8 | 0 | 0.0 | 0 | 0.0 | 0 | 0.0 | 0 | 0.0 | 0 | 0.0 | 0 | 0.0 |
| Combined Overweight | 0 | 0.0 | 3 | 14.3 | 0 | 0.0 | 5 | 23.8 | 0 | 0.0 | 2 | 9.5 | 0 | 0.0 | 3 | 14.3 | 0 | 0.0 | 2 | 9.5 |
| Total | 5 | 100.0 | 21 | 100.0 | 5 | 100.0 | 21 | 100.0 | 5 | 100.0 | 21 | 100.0 | 5 | 100.0 | 21 | 100.0 | 5 | 100.0 | 21 | 100.0 |
| **Age 16** | | | | | | | | | | | | | | | | | | | |  |
| **Underweight** | 4 | **2.3** | 5 | **2.0** | 11 | **6.2** | 31 | **12.3** | 11 | **6.2** | 31 | **12.3** | 5 | **2.8** | 5 | **2.0** | 9 | **5.1** | 9 | **3.6** |
| Normal | 135 | 76.7 | 219 | 86.9 | 103 | 58.2 | 166 | 65.9 | 131 | 74.0 | 196 | 77.8 | 135 | 76.3 | 222 | 88.1 | 135 | 76.3 | 219 | 86.9 |
| Overweight | 25 | 14.2 | 21 | 8.3 | 45 | 25.4 | 43 | 17.1 | 25 | 14.1 | 24 | 9.5 | 26 | 14.7 | 23 | 9.1 | 21 | 11.9 | 22 | 8.7 |
| Obese | 12 | 6.8 | 7 | 2.8 | 18 | 10.2 | 12 | 4.8 | 10 | 5.6 | 1 | 0.4 | 11 | 6.2 | 2 | 0.8 | 12 | 6.8 | 2 | 0.8 |
| **Combined Overweight** | 37 | **21.0** | 28 | **11.1** | 63 | **35.6** | 55 | **21.8** | 35 | **19.8** | 25 | **9.9** | 37 | **20.9** | 25 | **9.9** | 33 | **18.6** | 24 | **9.5** |
| Total | 176 | 100.0 | 252 | 100.0 | 177 | 100.0 | 252 | 100.0 | 177 | 100.0 | 252 | 100.0 | 177 | 100.0 | 252 | 100.0 | 177 | 100.0 | 252 | 100.0 |
| **Age 17** | | | | | | | | | | | | | | | | | | | |  |
| **Underweight** | 3 | **5.4** | 2 | **4.7** | 8 | **13.1** | 8 | **18.6** | 8 | **13.1** | 8 | **18.6** | 3 | **4.9** | 2 | **4.7** | 6 | **9.8** | 3 | **7.0** |
| Normal | 42 | 75.0 | 36 | 83.7 | 38 | 62.3 | 28 | 65.1 | 43 | 70.5 | 30 | 69.8 | 48 | 78.7 | 36 | 83.7 | 45 | 73.8 | 37 | 86.0 |
| Overweight | 7 | 12.5 | 5 | 11.6 | 9 | 14.8 | 6 | 14.0 | 9 | 14.8 | 5 | 11.6 | 6 | 9.8 | 5 | 11.6 | 6 | 9.8 | 3 | 7.0 |
| Obese | 4 | 7.1 | 0 | 0.0 | 6 | 9.8 | 1 | 2.3 | 1 | 1.6 | 0 | 0.0 | 4 | 6.6 | 0 | 0.0 | 4 | 6.6 | 0 | 0.0 |
| **Combined Overweight** | 11 | **19.6** | 5 | **11.6** | 15 | **24.6** | 7 | **16.3** | 10 | **16.4** | 5 | **11.6** | 10 | **16.4** | 5 | **11.6** | 10 | **16.4** | 3 | **7.0** |
| Total | 56 | 100.0 | 43 | 100.0 | 61 | 100.0 | 43 | 100.0 | 61 | 100.0 | 43 | 100.0 | 61 | 100.0 | 43 | 100.0 | 61 | 100.0 | 43 | 100.0 |
| Age 18 and above | | | | | | | | | | | | | | | | | | | |  |
| Underweight | 0 | 0.0 | 0 | 0.0 | 0 | 0.0 | 1 | 33.3 | 0 | 0.0 | 1 | 33.3 | 0 | 0.0 | 0 | 0.0 | 0 | 0.0 | 1 | 33.3 |
| Normal | 7 | 77.8 | 3 | 100.0 | 7 | 77.8 | 2 | 66.7 | 8 | 88.9 | 2 | 66.7 | 8 | 88.9 | 3 | 100.0 | 8 | 88.9 | 2 | 66.7 |
| Overweight | 1 | 11.1 | 0 | 0.0 | 1 | 11.1 | 0 | 0.0 | 0 | 0.0 | 0 | 0.0 | 0 | 0.0 | 0 | 0.0 | 0 | 0.0 | 0 | 0.0 |
| Obese | 1 | 11.1 | 0 | 0.0 | 1 | 11.1 | 0 | 0.0 | 1 | 11.1 | 0 | 0.0 | 1 | 11.1 | 0 | 0.0 | 1 | 11.1 | 0 | 0.0 |
| Combined Overweight | 2 | 22.2 | 0 | 0.0 | 2 | 22.2 | 0 | 0.0 | 1 | 11.1 | 0 | 0.0 | 1 | 11.1 | 0 | 0.0 | 1 | 11.1 | 0 | 0.0 |
| Total | 9 | 100.0 | 3 | 100.0 | 9 | 100.0 | 3 | 100.0 | 9 | 100.0 | 3 | 100.0 | 9 | 100.0 | 3 | 100.0 | 9 | 100.0 | 3 | 100.0 |
